# Human Adipose-derived Pericytes Display Steroidogenic Lineage Potential *in Vitro* and Influence Leydig Cell Regeneration *in Vivo* in Rats

**DOI:** 10.1038/s41598-019-50855-0

**Published:** 2019-10-21

**Authors:** Michael Curley, Zaniah N. Gonzalez, Laura Milne, Patrick Hadoke, Ian Handel, Bruno Péault, Lee B. Smith

**Affiliations:** 1 MRC Centre for Reproductive Health, University of Edinburgh, The Queen’s Medical Research Institute, 47 Little France Crescent, Edinburgh, EH16 4TJ United Kingdom; 2 MRC Centre for Regenerative Medicine, University of Edinburgh, Edinburgh Bioquarter, 5 Little France Drive, Edinburgh, EH16 4UU United Kingdom; 3 The British Heart Foundation Centre for Cardiovascular Science, University of Edinburgh, The Queen’s Medical Research Institute, Edinburgh, EH16 4TJ United Kingdom; 4 The Roslin Institute and Royal (Dick) School of Veterinary Studies, University of Edinburgh, Edinburgh, EH25 9RG United Kingdom; 5 Department of Orthopaedic Surgery and Broad Stem Cell Center, University of California at Los Angeles, 615 Charles E Young Dr S, Los Angeles, CA 90095 USA; 6 School of Environmental and Life Sciences, University of Newcastle, Callaghan, NSW 2308 Australia

**Keywords:** Reproductive biology, Regeneration

## Abstract

Exogenous androgen replacement is used to treat symptoms associated with low testosterone in males. However, adverse cardiovascular risk and negative fertility impacts impel development of alternative approaches to restore/maintain Leydig cell (LC) androgen production. Stem Leydig cell (SLC) transplantation shows promise in this regard however, practicality of SLC isolation/transplantation impede clinical translation. Multipotent human adipose-derived perivascular stem cells (hAd-PSCs) represent an attractive extragonadal stem cell source for regenerative therapies in the testis but their therapeutic potential in this context is unexplored. We asked whether hAd-PSCs could be converted into Leydig-like cells and determined their capacity to promote regeneration in LC-ablated rat testes. Exposure of hAd-PSCs to differentiation-inducing factors *in vitro* upregulated steroidogenic genes but did not fully induce LC differentiation. *In vivo*, no difference in LC-regeneration was noted between Sham and hAd-PSC-transplanted rats. Interestingly, *Cyp17a1* expression increased in hAd-PSC-transplanted testes compared to intact vehicle controls and the luteinising hormone/testosterone ratio returned to Vehicle control levels which was not the case in EDS + Sham animals. Notably, hAd-PSCs were undetectable one-month after transplantation suggesting this effect is likely mediated *via* paracrine mechanisms during the initial stages of regeneration; either directly by interacting with regenerating LCs, or through indirect interactions with trophic macrophages.

## Introduction

Establishment and maintenance of the male phenotype requires adequate production and action of androgens (e.g. testosterone) which are primarily produced by Leydig cells located in the testicular interstitium. Further to their well-established role in male reproductive development and function, increasing evidence suggests androgens also support overall general health in men as low circulating testosterone levels are associated with chronic cardio-metabolic disorders^1–5^. Androgen replacement therapy is commonly used to treat symptoms associated with lower circulating testosterone both in ageing men with late-onset hypogonadism and in young hypogonadal males. However, exogenous testosterone replacement disrupts the hypothalamic-pituitary-gonadal (HPG) axis compromising fertility^6,7^ - a potentially catastrophic side effect for younger men wishing to father children. Moreover, the risks and benefits of androgen replacement therapy, particularly in older men, are unclear with a number of clinical studies reporting adverse cardiovascular events associated with exogenous testosterone administration^8–10^. As such, novel therapies which promote and/or maintain endogenous HPG axis function, keeping testicular testosterone production within a normal physiological range, may be more appropriate to improve Leydig cell function in cases of primary hypogonadism. Ideally, a single long-lasting therapeutic intervention would likely be more cost-effective compared to repeated exogenous testosterone replacement however, overcoming time and cost associated with initial steps of *in vitro* expansion/manipulation of stem cells populations remain a significant challenge.

The identity and behaviour of the stem cells that give rise to testosterone-producing Leydig cells within the testicular interstitium has been an area of intense research - particularly in relation to harnessing their regenerative properties as an alternative to exogenous androgen replacement. Stem Leydig cells have been prospectively isolated from rodent and human testes and extensively characterised both *in vitro* and in *in vivo* transplantation models^11–14^. Although these studies have significantly enhanced our understanding of stem Leydig cell differentiation, extraction of stem cells from a patient’s testis may be impractical – potentially limiting their utility as a regenerative cell therapy. As such, identification of a suitable extra-gonadal stem cell source is required. Whilst the precise origin of stem Leydig cells within the testis is debated, with both peritubular^15^ and perivascular^16^ origins proposed; Davidoff *et al*. suggested that, following EDS-mediated Leydig cell ablation in rats, Leydig cells regenerate from perivascular stem cells (PSCs) which express neural/glial antigen 2 (NG2) and platelet-derived growth factor receptor-beta (PDGFRβ)^16^. Interestingly, a universal identity for multipotent stem cells residing in the perivascular niche in multiple tissues has been proposed. Specifically, PSCs are identifiable by NG2, PDGFRβ and CD146 (cluster of differentiation 146) expression and they also express as a number of ‘mesenchymal stem cell’ markers including CD90^17^. These markers also identify stem Leydig cells in the testis^12,16,18^, raising the possibility that extra-gonadal PSCs may represent a viable option for use in autologous regenerative cell therapies to promote Leydig cell function.

Adipose tissue is a well-endowed source of PSCs and is easily accessible with minimally invasive techniques. Mouse adipose-derived stem cells have been driven down the steroidogenic lineage *in vitro* following transduction with a steroidogenic factor-1 (SF1) expressing adenovirus^19^. However, the resulting cells favourably produced glucocorticoids over androgens suggesting additional factors are required to obtain functional Leydig-like cells. In an experimentally induced ageing model, intravenous injection of rat adipose-derived stem cells were reported to alleviate testicular dysfunction although the mechanism is unclear^20^. The regenerative properties of human adipose-derived perivascular stem cells (hAd-PSCs; CD146^pos^, CD34^neg^, CD31^neg^, CD45^neg^), acting *via* direct and paracrine mechanisms, have been recognised in orthopaedic research models^21–24^. However, the regenerative potential of hAd-PSCs to promote Leydig cell function in the testis has not been explored. Specifically, whether hAd-PSCs can be transformed into Leydig-like cells *in vitro* and/or *in vivo* and if they can support endogenous Leydig cell regeneration/function is unknown.

To address this, we exposed hAd-PSC cultures to a predefined combination of hormones and growth factors known to induce *in vitro* differentiation of human and rodent stem Leydig cells. Additionally, we transplanted hAd-PSCs cultured with or without differentiation inducing factors into Leydig cell-ablated rat testes and monitored Leydig cell regeneration over 35 days. This revealed that whilst hAd-PSCs may harbour some steroidogenic lineage potential *in vitro*, they do not directly contribute to the regenerating Leydig cell population *in vivo*. Instead, hAd-PSCs may promote endogenous Leydig cell regeneration and increase the size of the newly formed Leydig cell population.

## Results

### Steroidogenic Potential of hAd-PSCs *in vitro*

**Figure 1:**
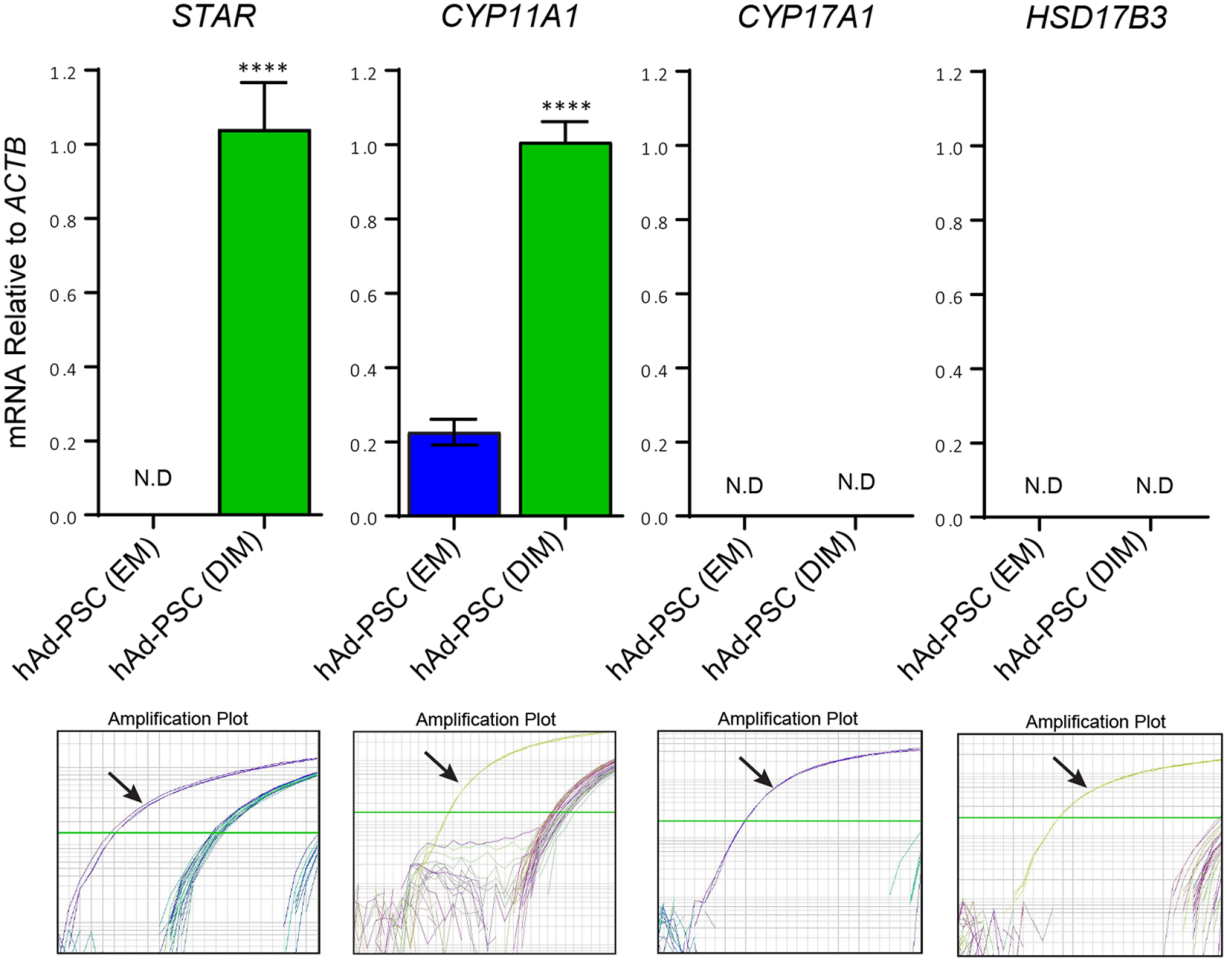
Induction of steroidogenic *mRNA* expression in hAd-PSCs cultured in differentiation inducing medium. Expression of *STAR* (steroidogenic acute regulatory protein) and *CYP11A1* (P450 cholesterol side-chain cleavage enzyme) was induced in human adipose-derived perivascular stem cells (hAd-PSCs) after one week culture in differentiation inducing media (DIM; *t*-test; ****p =  < 0.0001). Neither *CYP17A1* (17α-hydroxylase, 17,20-lyase) nor *HSD17B*3 (hydroxysteroid dehydrogenase 17-beta type 3) were detectable. Arrows in bottom panels indicate the amplification adult human testis cDNA as a positive control. Data presented are mean ± SEM from n = 6 separate wells per group. N.D = Not detected.

Human adipose-derived perivascular stem cells are multipotent stem/progenitor cells with myogenic, osteogenic and adipogenic properties^21,22,25,26^. However, the steroidogenic potential of hAd-PSCs has not been reported. Therefore, we first asked whether hAd-PSCs could be transformed into steroidogenic Leydig-like cells *in vitro*. hAd-PSCs were cultured in a differentiation inducing media (DIM) containing luteinising hormone (LH), platelet-derived growth factor beta (PDGF-BB), insulin-like growth factor-1 (IGF-1) and thyroid hormone (T3). This combination of trophic factors can stimulate differentiation of rodent and human stem Leydig cells *in vitro*^11–14^. As such, we reasoned it could induce the steroidogenic lineage in extra-gonadal hAd-PSCs. After one week of culture, cells were harvested and the *mRNA* expression of genes involved in androgen biosynthesis was measured by qRT-PCR and compared to control cells cultured in expansion media only (EM; DMEM GlutaMAX™/fetal bovine serum). Exposure of hAd-PSCs to DIM induced the expression of *STAR* and *CYP11A1* (Fig. 1), encoding the steroidogenic acute regulatory protein and P450 cholesterol side-chain cleavage enzyme which function in the initial and rate-limiting steps of steroidogenesis. Conversely, neither *CYP17A1* nor *HSD17B3*, encoding the enzymes 17α-hydroxylase, 17,20-lyase and hydroxysteroid dehydrogenase 17-beta type 3 required for the final steps of the classical androgen biosynthetic pathway, were detected in EM or DIM cultured hAd-PSCs (Fig. 1). These data suggest that, whilst hAd-PSCs can be driven to differentiate towards a steroidogenic lineage, these specific *in vitro* conditions are insufficient to convert them into fully functional Leydig-like cells.

### hAd-PSCs are not Incorporated into the Newly Formed Leydig Cell Population *in Vivo*

**Figure 2:**
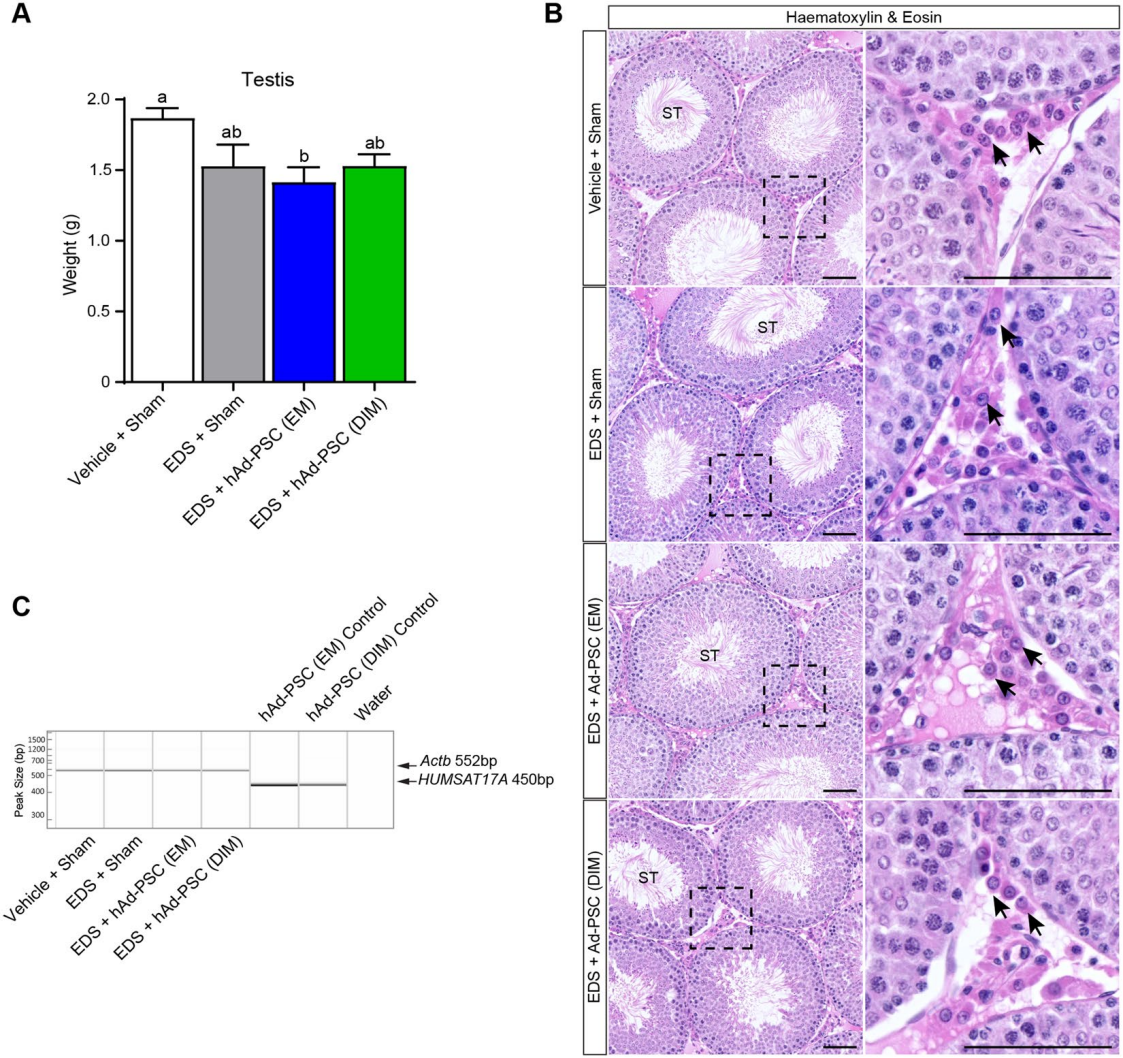
Effect of hAd-PSCs on testis weight and histology. (**A**) Testis weight recovered to Vehicle + Sham control values in EDS + Sham and EDS + hAd-PSC (DIM) groups but remained significantly lower in the EDS + hAd-PSC (EM) group (Kruskal-Wallis; *p* = 0.0264). Means were compared between groups using Dunn’s post hoc analysis. Groups that share a letter superscript are not significantly different. Data presented are mean ± SEM from n = 5–6 animals per group. (**B**) Representative H&E stained testis sections revealed testicular architecture was largely normal 35 days post Leydig cell ablation. Full spermatogenesis within seminiferous tubules (ST) and abundant interstitial Leydig cells (arrows) were observed in all groups. Scale = 100 µm. **(C)** Multiplex genomic PCR for human and rat specific genomic DNA (*HUMSAT17A*; 450 bp and *Actb*; 552 bp respectively) in testis biopsies 35 days after transplantation (EDS + 35). No human DNA was detected in either hAd-PSC (EM) or hAd-PSC (DIM) transplanted testes. Genomic DNA isolated directly from EM and DIM hAd-PSC cultures was included as a positive control.

The full complement of factors that control adult Leydig cell development *in vivo* is yet to be defined. As such, derivation of functional Leydig-like cells from hAd-PSCs likely requires additional critical mediators of Leydig cell development. To determine whether unknown trophic factors could complete the transformation of hAd-PSCs to Leydig-like cells, we transplanted either EM or DIM cultured hAd-PSCs into the interstitial compartment of the rat testis 4 days after EDS-mediated Leydig cell ablation (i.e. into a *bona fide* environment conducive to Leydig cell development). When animals were sacrificed 35 days after EDS treatment, no difference in body weight was observed between groups, suggesting neither EDS nor hAd-PSCs had major negative systemic side effects (Supplemental Fig. 1). Recovery of testis weight to that of Vehicle + Sham controls was observed in the EDS + Sham and EDS + hAd-PSC (DIM) groups. Although there was no significant difference in testis weight between any of the EDS-treated groups, EDS + Ad-PSC (EM) testes remained significantly lighter than Vehicle + Sham controls at 35 days post EDS which may represent delayed recovery of spermatogenesis following transient androgen deprivation in this group (Fig. 2A).

Testicular histology was grossly similar between groups at 35 days post EDS, with complete spermatogenesis taking place within seminiferous tubules, and abundant interstitial Leydig cells (Fig. 2B). However, in EDS + hAd-PSC (EM) and EDS + hAd-PSC (DIM) groups, occasional regions containing atrophic seminiferous tubules were observed (Supplemental Fig. 2). As this was not observed in the EDS + Sham control group, it may be a consequence of an immune response to the xenogenic hAd-PSCs rather than a due to surgical procedures or EDS toxicity. When genomic DNA (gDNA) was isolated from control and hAd-PSC transplanted testes and interrogated for the presence of human-specific gDNA (*HUMSAT17A*), no human gDNA was detected either in EM or in DIM hAd-PSC transplanted testes (Fig. 2C). While we cannot rule out a transient transformation of hAd-PSCs into Leydig-like cells at earlier time-points, these data suggest that they are not incorporated into, and retained within, the endogenous Leydig cell population in this xenogeneic transplantation model.

### Effect of hAD-PSCs on Endogenous Leydig cell Regeneration and Function *in vivo*

**Figure 3:**
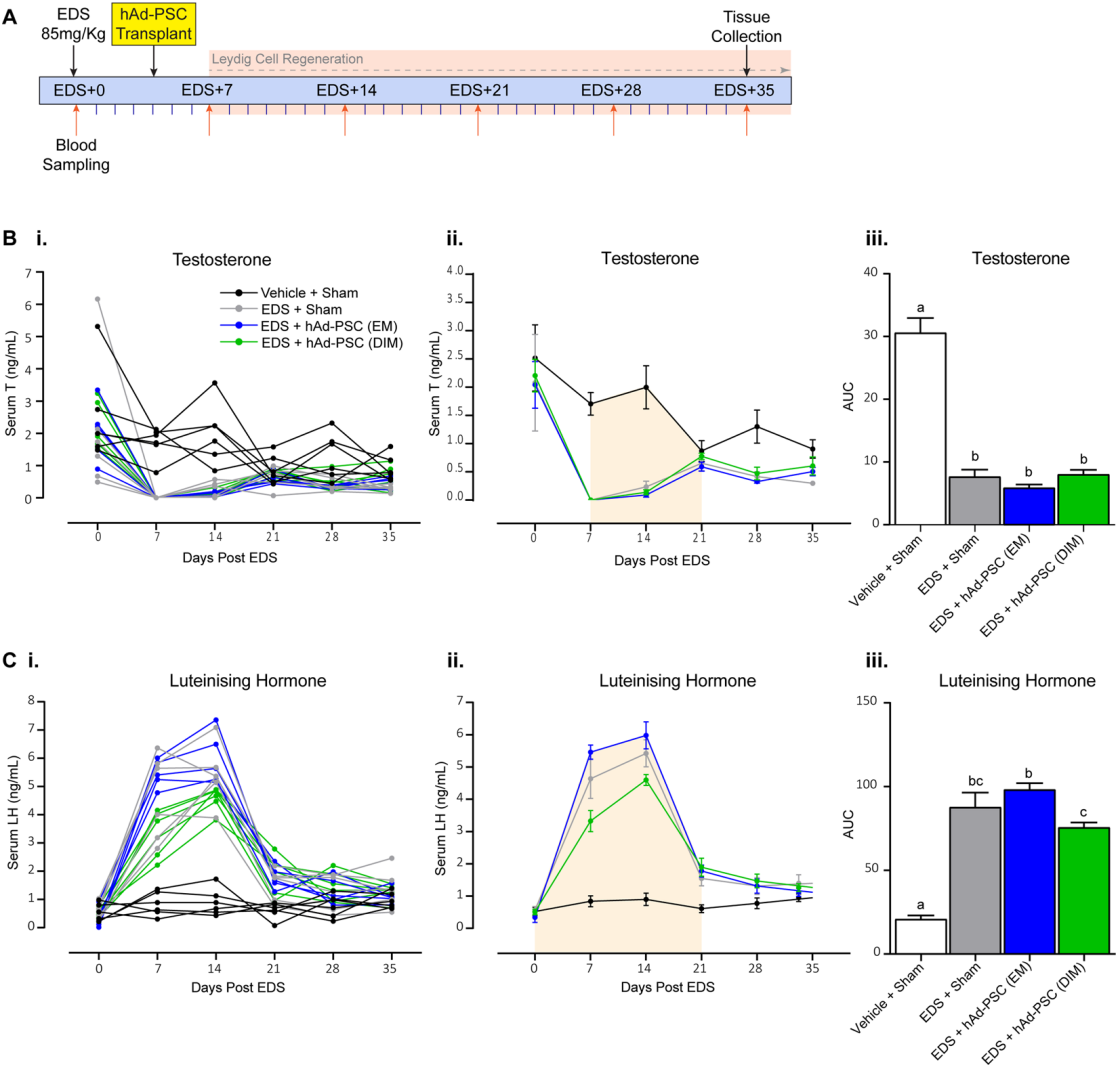
Circulating hormone measurements following Leydig cell ablation and hAd-PSC transplantation. (**A**) Leydig cells were ablated using a single dose of ethane dimethanesulphonate (EDS). Human perivascular stem cells (hAd-PSCs), cultured in expansion medium (EM) or differentiation inducing medium (DIM), were transplanted into the testis interstitium 4 days after EDS. Circulating hormones were measured in sera from tail vein bleeds collected weekly until animals were sacrificed after 35 days (EDS + 35). Both individual animals (**i**) and the mean ± SEM for each group (**ii**) are plotted. Circulating testosterone (**B**) was undetectable in EDS-treated animals after 7 days and returned to Vehicle + Sham control levels after 21 days. Area under the curve (AUC) analysis (**Biii**) revealed no difference in recovery of circulating testosterone levels in animals transplanted with either EM or DIM treated hAd-PSCs compared to EDS + Sham controls. Circulating luteinising hormone (**C**) increased following EDS administration, peaking at 14 days, and returned to Vehicle + Sham control levels after 28 days. AUC (**Ciii**) was significantly lower in the hAd-PSC (DIM) group compared to the hAd-PSC (EM) group. 1-way ANOVAs were used to identify differences between groups (*p* =  < 0.0001 for both testosterone and LH). Tukey’s post-hoc analysis was used to compare means between groups where a shared letter denotes no significant difference. The orange shaded areas in (ii) highlight the proportion of the data used for area under the curve (AUC, iii) analyses based on the time taken for testosterone to return to Vehicle + Sham control levels in EDS + Sham animals (EDS + 21). Data presented are mean ± SEM from n = 5–6 separate animals per group.

Complete Leydig cell ablation occurs within 3–7 days following EDS administration, followed by regeneration of the endogenous Leydig cell population and restoration of circulating testosterone levels within 3–6 weeks^27,28^. To monitor Leydig cell development in control and hAD-PSC transplanted testes, serial measurement of circulating testosterone (T) and luteinising hormone (LH) concentrations was performed (Fig. 3A). Testosterone was undetectable 7 days after EDS treatment but returned to control levels by 21 days (Fig. 3B), confirming successful Leydig cell ablation and regeneration. To determine the impact of the transplanted hAd-PSCs on recovery of circulating testosterone between these time points, area under the curve (AUC) was calculated. As expected, AUC was significantly decreased in all EDS treated groups compared to Vehicle + Sham controls. However, no difference was observed when EM or DIM cultured hAd-PSCs were transplanted into the testis (Fig. 3B). In line with the circulating testosterone profile, an increase in LH, peaking at 14 days, was observed following EDS treatment (Fig. 3C). AUC analysis (0–21 days post EDS) revealed that the LH spike, induced by removal of testosterone negative feedback following Leydig cell ablation, was significantly blunted in the hAd-PSC (DIM) group compared to the hAd-PSC (EM) group (Fig. 3C) although not significantly different from EDS + Sham controls.

**Figure 4:**
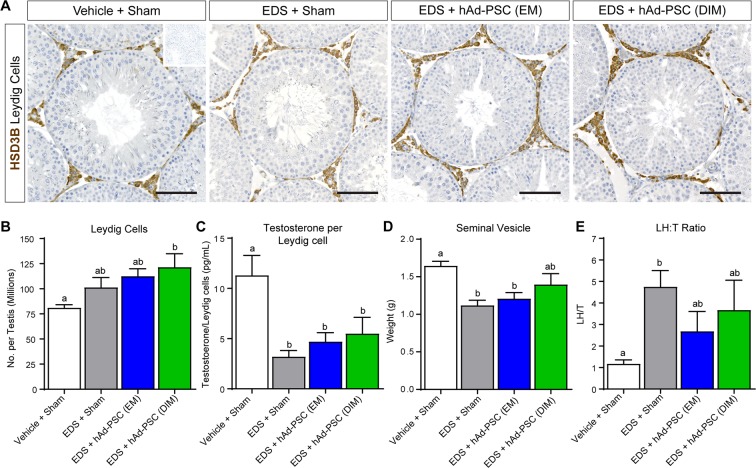
Effect of hAd-PSCs on Leydig cell number and function. (**A**) Immunostaining of hydroxysteroid dehydrogenase 3-beta (HSD3B) marking testicular Leydig cells 35 days after ethane dimethanesulphonate (EDS)-mediated Leydig cell ablation with or without transplantation of hAd-PSCs cultivated in either expansion medium (EM) or differentiation inducing medium (DIM). Inset = primary antibody negative control. Scale bars = 100 µm. (**B**) Stereological analysis revealed a significantly increased number of Leydig cells in testes transplanted with hAd-PSCs (DIM) compared to Vehicle + Sham controls (1-way ANOVA; *p* = 0.0204). (**C**) Circulating testosterone relative to Leydig cell number was reduced in all three experimental groups compared to Vehicle + Sham controls (1-way ANOVA; *p* = 0.0035). However, seminal vesicle weight (**D**) as a marker of peripheral androgen action was recovered to Vehicle + Sham control levels in the hAd-PSC (DIM) group (1-way ANOVA; *p* = 0.0044). (**E**) The luteinising hormone:testosterone ratio remained significantly elevated in the EDS + Sham group but not in either hAd-PSC transplanted group compared to Vehicle + Sham controls (Kruskal-Wallis; *p* = 0.0083). For one-way ANOVA, means were compared between groups using Tukey’s post hoc analysis. For Kruskal-Wallis, means were compared between groups using Dunn’s post hoc analysis. In each case, groups that share a letter superscript are not significantly different. Data presented are mean ± SEM from n = 5–6 animals per group.

**Figure 5:**
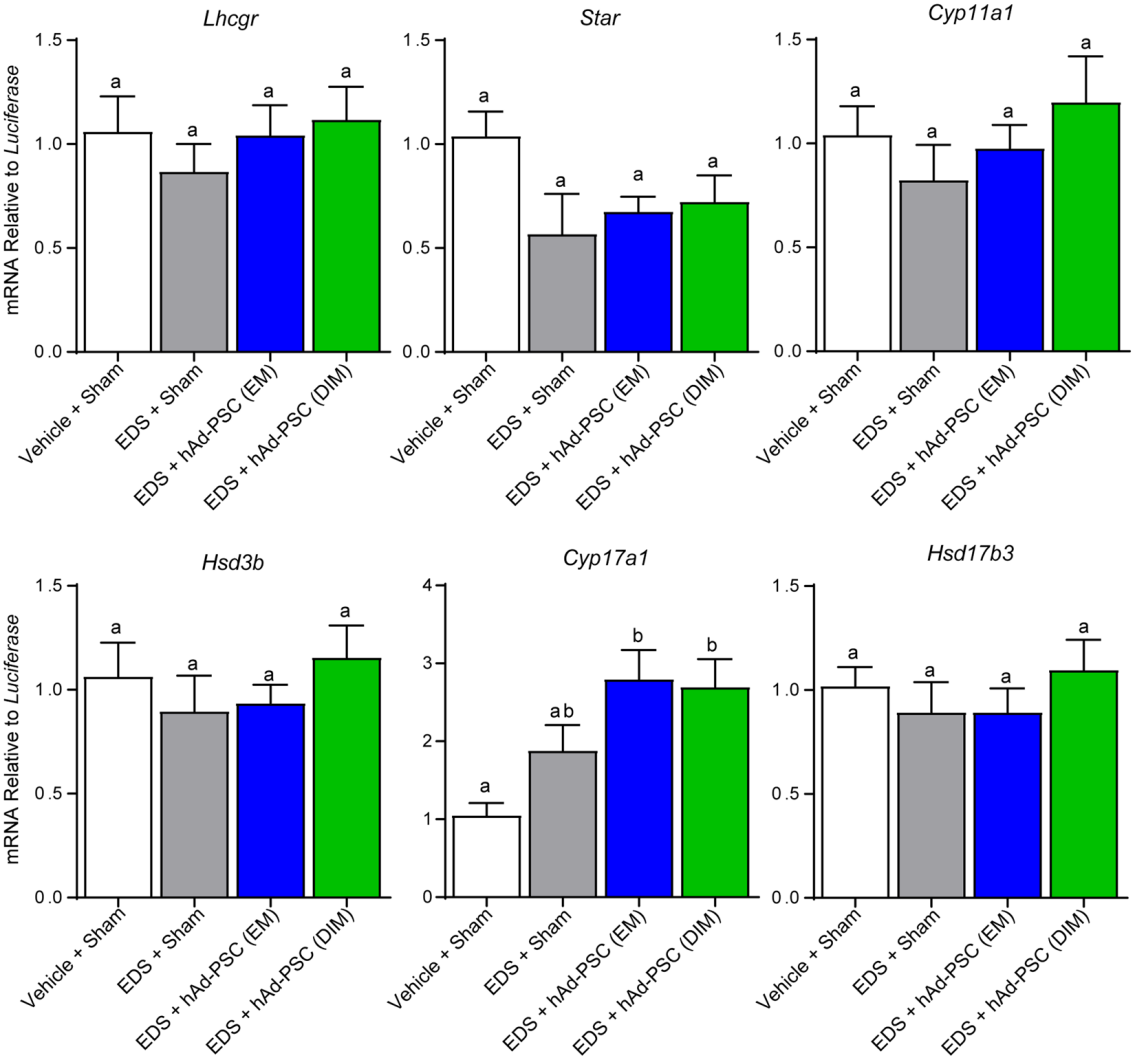
Leydig cell steroidogenic *mRNA* expression. No difference in *mRNA* expression levels of luteinising hormone/chorionic gonadotropin receptor (*Lhcgr*); steroidogenic acute regulatory protein (*Star*); P450 cholesterol side-chain cleavage enzyme (*Cyp11a1*); hydroxysteroid dehydrogenase 3-beta (*Hsd3b*); or hydroxysteroid dehydrogenase 17-beta type 3 (*Hsd17b3*) was noted between experimental groups and Vehicle + Sham controls (1-way ANOVA). However, expression of 17α-hydroxylase, 17,20-lyase (*Cyp17a1*) was significantly increased both in hAd-PSC (EM) and in hAd-PSC (DIM) groups (1-way ANOVA; *p* = 0.0025). Tukey’s post-hoc analysis was used to compare means between groups. Groups that share a letter superscript are not significantly different. Data presented are mean ± SEM from n = 5–6 separate animals per group.

We next asked whether Leydig cell number and/or function was altered in testes transplanted with either EM or DIM cultured hAd-PSCs. When assessed 35 days after EDS-mediated Leydig cell ablation, positive immunostaining for the Leydig cell marker hydroxysteroid dehydrogenase 3-beta (HSD3B) was observed in the interstitial compartment of Vehicle + Sham and EDS + Sham control testes as expected. Positive HSD3B staining was also observed both in EM and in DIM hAd-PSC transplanted testes (Fig. 4A). Surprisingly, stereological analysis revealed a significant increase in the number of Leydig cells in hAd-PSC (DIM) testes compared to Vehicle + Sham controls (Fig. 4B). Although we noted no significant differences between hAd-PSC and EDS + sham groups, Leydig cell numbers in EDS + Sham and EDS + hAd-PSC (EM) were not significantly increased compared to Vehicle + Sham Controls. Circulating testosterone relative to Leydig cell number was reduced in EDS + Sham and both hAd-PSC (EM) and (DIM) transplanted groups (Fig. 4C). However, seminal vesicle weight (as a readout of peripheral androgen action^29–31^) recovered to the equivalent of Vehicle + Sham controls only in the hAd-PSC (DIM) group although no differences were noted between EDS + Sham and hAd-PSC-transplanted groups. Additionally, the LH/T ratio (as an indicator of primary Leydig cell function) remained significantly elevated in the EDS + Sham group, whereas no difference was noted between EM or DIM hAd-PSC and Vehicle + Sham control groups (Fig. 4E). This suggests that the function of the newly formed Leydig cell population may be improved when hAd-PSCs are transplanted into the testis interstitium following EDS-mediated Leydig cell ablation. To further probe Leydig cell function, we measured *mRNA* expression levels of luteinising hormone/chorionic gonadotrophin receptor (*Lhcgr*) and steroidogenic acute regulatory protein (*Star*) as well as cytochrome P450 and hydroxysteroid dehydrogenase enzymes required for the conversion of cholesterol to testosterone in Leydig cells (*Cyp11a1, Cyp17a1* and *Hsd3b1, Hsd17b3* respectively). No difference in the expression of *Lhcgr, Star, Cyp11a1, Hsd3b1* or *Hsd17b3* was noted between control and hAd-PSC transplanted testes. However, a significant increase in *Cyp17a1* expression (required for the conversion progestagens to androgens) was observed both in (EM) and in (DIM) hAd-PSC transplanted testes (Fig. 5). This pattern of expression was similar when data were corrected for Leydig cell number (Supplemental Fig. 3) and therefore reflects an alteration in Leydig cell function, rather than simply resulting from an increase in Leydig cell number. Together, these data indicate that hAd-PSCs pre-exposed to differentiating-inducing factors may promote primary function and, intriguingly, the final size of the regenerating Leydig cell population.

## Discussion

The current strategy for treating hypogonadism is the administration of exogenous testosterone. However, this approach can have negative side effects including adverse cardiovascular events^8–10^ and disruption of normal spermatogenesis which may not be desirable if maintenance of fertility is required^6,7^. In recent years, efforts have been focussed on developing stem cell-based regenerative therapies to restore endogenous androgen production as an alternative to exogenous testosterone replacement^32^. Ultimately, a single, long-lasting therapeutic intervention could prove more cost-effective than repeated testosterone replacement. In the current study, we asked whether human adipose-derived perivascular stem cells (hAd-PSC) could be converted into testosterone producing Leydig-like cells. We also assessed the regenerative capacity of hAd-PSCs to promote endogenous testosterone production in an *in vivo* rat model of Leydig cell ablation-regeneration. Our results demonstrate that hAd-PSCs harbour some steroidogenic lineage potential *in vitro* but they do not directly contribute to the regenerating Leydig cell population *in vivo*. Instead, hAd-PSCs may influence endogenous Leydig cell regeneration, increasing the size of the newly formed Leydig cell population perhaps *via* paracrine mechanisms during the initial stages of regeneration. However, whether autologous hAd-PSCs could be isolated and directly transplanted back into the testis, bypassing the time and cost of *in vitro* expansion and/or differentiation, remains to be tested.

In 2006, Ge and colleagues described a cocktail of hormones and growth factors sufficient for the *in vitro* generation of testosterone-producing Leydig-like cells from platelet derived growth factor alpha-positive (PDFGR-α) stem cells isolated from neonatal rat testes. More recently, this differentiation inducing media (DIM) has been used to generate testosterone producing Leydig-like cells both from nestin-positive and CD51-positive stem cell populations isolated from neonatal and adult mouse testes respectively^12,13^. In each of these studies, induction of steroidogenic cell lineage markers including SF-1, GATA-4, LHCGR, STAR, CYP11A1, HSD3B6 and CYP17A1 was reported after 7–11 days of culture in DIM. Whilst none of the above studies demonstrated the expression of HSD17B3 – the enzyme required for the final step of classical testosterone biosynthesis – all reported testosterone production by cells cultured in DIM, suggesting a successful transformation of the respective stem cell populations into functional Leydig-like cells. In the present study, we were able to detect the expression of *STAR* and *CYP11A1* in cultures of hAd-PSCs exposed to DIM for 7 days however, neither *CYP17A1* nor *HSD17B3* were detectable. As such, whilst hAd-PSCs exposed to DIM may acquire some steroidogenic potential, these specific *in vitro* conditions are not sufficient to convert them into functional testosterone producing cells. This could be attributed to divergent responsiveness of rodent and human stem cells to DIM. However, Leydig-like cells have been generated from human p75-positive testicular stem cells following DIM exposure; albeit after a longer incubation of 28 days^14^. As such, it is likely that additional experimental factors may preclude complete transformation of hAd-PSCs into functional Leydig-like cells. For example, the expansion media utilised in previous studies contained additional supplements including dexamethasone, leukemia inhibitory factor, platelet derived growth factor-beta, fibroblast growth factor and epidermal growth factor^11–14^. These factors have been implicated in aspects of Leydig cell development and function^28,33–40^ and thus, may ‘prime’ the cells for steroidogenic lineage differentiation prior to DIM exposure. Additionally, significant heterogeneity exists between stem cells isolated from different tissues, or even between stem cell sub-populations isolated from the same tissue^26,41–44^. Therefore, whilst hAd-PSCs possess multi-lineage potential^25^, stem cell subsets isolated from testis tissue may have increased propensity to develop into Leydig cells. This requires further investigation. Interestingly, we observed *CYP11A1* expression in hAd-PSCs cultured in EM only, albeit at a lower level than DIM-cultured cells. At present, we cannot conclude whether EM specifically induces the expression of *CYP11A1* in hAd-PSCs or if endogenous hAd-PSCs express *CYP11A1* prior to isolation and culture. The extent to which stem cell-like behaviours of pericytes, or indeed other ‘stem cells’ are an artefact of *ex vivo* and *in vitro* manipulations is not entirely clear. Thus, we use the term ‘stem cell’ with caution^45^. Recently, it has been suggested that, in adult mice, endogenous pericytes in multiple organs do not behave as stem cells *in vivo*^46^. Therefore, hAd-PSCs may only possess limited stem cell-like properties rather than being true ‘stem cells’ capable of Leydig cell differentiation.

Testicular development and function is tightly regulated by a complex paracrine signalling network and the full complement of factors that control adult Leydig cell development *in vivo* is yet to be defined. Furthermore, the majority of our understanding comes from rodent models and thus, we may underappreciate the requirements for human adult Leydig cell development. Nevertheless, we reasoned that transplantation into the rat testis interstitium during Leydig cell regeneration (following EDS-mediated Leydig cell ablation) would expose hAd-PSCs to additional trophic factors and possibly promote differentiation into Leydig-like cells. In general, no differences in Leydig cell regeneration were noted between Sham and hAd-PSC-transplanted groups following EDS exposure. Neither EM nor DIM cultured hAd-PSCs appeared to affect the eventual recovery of circulating testosterone levels and transplanted hAd-PSCs were not incorporated into the regenerating Leydig cell population. Previous studies have demonstrated that the size newly regenerated Leydig cell population following EDS treatment is equal to intact Vehicle controls 5-weeks post EDS injection in Wistar rats^47^. In the present studies, transplantation of DIM cultured hAd-PSCs increased endogenous Leydig cell number above Vehicle + Sham animals and, seminal vesicle weight (as a measure of peripheral androgen action) was recovered equivalent to Vehicle + Sham controls which was not observed either in EDS + Sham or in EDS + hAd-PSC (EM) animals. Furthermore, in hAd-PSC transplanted animals, *Cyp17a1* expression (required for the conversion of progestagens to androgens) was increased following EDS exposure compared to Vehicle + Sham controls and the luteinising hormone/testosterone ratio (as a measure of primary Leydig cell function) returned to Vehicle + Sham control levels. In each case, this was not observed in EDS + Sham animals and may be a subtle indicator of altered Leydig cell regeneration kinetics in hAd-PSC-transplanted testes. Whether this is a direct attribute of hAd-PSCs or is simply an adjuvant-type response in the testicular microenvironment requires further investigation. Interestingly, accelerated adult Leydig cell development and increased *Cyp17a1* expression has been described in fetal Leydig cell-ablated rat testes^48^. Additionally, injection of FGF16 into the rat testis interstitium following Leydig cell ablation stimulates stem and immature Leydig cell proliferation resulting expansion of the adult Leydig cell population^49^. These studies highlight a role for the interstitial microenvironment in controlling adult Leydig cell development/number and support our hypothesis that alterations in the testis microenvironment induced by hAd-PSCs may influence Leydig cell population size. It is noteworthy that testicular macrophages are intimately involved in Leydig cell development and function^50–52^. Following EDS treatment, testicular macrophages are activated to clear apoptotic Leydig cells from the interstitium^53,54^ and it has been previously documented that testicular macrophages influence Leydig cell response to pituitary gonadotrophin^51,55^. As such, enhanced macrophage activation following introduction of xenogenic hAd-PSCs into Leydig cell-ablated testes may explain the changes in Leydig cell number/function observed in hAd-PSC-transplanted rats. This requires further investigation.

A number of studies have reported successful engraftment and differentiation of rodent stem Leydig cells following EDS mediated Leydig cell ablation in immunocompetent hosts^11–13^. However, in these studies testes were analysed 10–12 days after transplantation. Therefore, whether the cells could survive longer term engraftment is unclear. In the current study, we were unable to detect hAd-PSCs in transplanted testes 1 month after injection, indicating that they do not survive long term in the rat testis. This is consistent with previous reports of hAd-PSCs in a mouse model of ischaemic heart injury^56^. Lineage tracing of GFP labelled hAd-PSCs transplanted into the myocardium demonstrated that only a very small number of cells differentiated into cardiac cells; suggesting the positive effects of hAd-PSCs on cardiac recovery were likely mediated by a paracrine mecahnism^56^. Similarly, adipose-derived stem cells were reported to have positive effects in a rat model of tobacco-induced erectile dysfunction by reducing oxidative stress. EdU (5-ethynyl-2′-deoxyuridine) labelling of transplanted cells revealed minimal engraftment four weeks after injection leading the authors to speculate that paracrine release of cytokines/growth factors may be responsible for the therapeutic effect^57^. Indeed, perivascular stem cells secrete a variety of factors including PDGF-BB^58^ and leukemia inhibitory factor (LIF)^56^, both of which are thought to play a role in stem Leydig cell proliferation/differentiation^28,38^. Furthermore, pre-incubation of primary rat Leydig cells with PDGF-BB significantly enhances LH-stimulated testosterone synthesis^59^.

Previous studies have demonstrated that macrophage number increases 2–3 fold to clear apoptotic Leydig cells following EDS treatment^54,55^ and, when testicular macrophages are depleted, Leydig cells do not fully regenerate^52^ highlighting an important role of trophic macrophages in the Leydig cell regeneration process. Additionally, increasing macrophage number in the testis has been shown to reduce LH levels without changing testosterone levels suggesting a systemic as well as local effect of macrophages on Leydig cell function. It is therefore conceivable that transplantation of xenogenic hAd-PSCs into the rat testis may have enhanced the ongoing inflammatory process in the EDS-exposed testes and thus impact the initial stages of Leydig cell regeneration. We did notice *foci* containing atrophic seminiferous tubules in hAd-PSC-transplanted testes 35-days after EDS-mediated Leydig cell ablation which may be indicative of an aberrant inflammatory response to xenogenic hAd-PSCs as this was not observed in EDS + Sham testes. However, as we sacrificed animals only at 35-days post EDS, we could not fully evaluate this possibility. As such, the full extent to which hAd-PSCs influence Leydig cell regeneration and identification of the precise mechanisms (direct or indirect) by which they influence Leydig cell population size requires further study.

We acknowledge that our studies used a relatively small number of immunocompetent animals without immunosuppression, which may have enhanced the inflammatory response to Leydig cell death post EDS treatment due to xenogeneic rejection. However, previous studies of xenogenic transplantation in immuno-deficient models also noted limited survival and differentiation of transplanted stem cells, despite positive effects on tissue regeneration^56,60,61^. Additionally, the hAd-PSCs used in this study were derived from a single donor which may confound our assessment of the regenerative capacity of hAd-PSCs as donor age and sex may influence hAd-PSC activity^62^. Nevertheless, these studies are, to the best of our knowledge, the first description of human Ad-PSCs in an *in vivo* model of Leydig cell injury. We suggest that transplantation of DIM-exposed hAd-PSCs into the Leydig cell-ablated testis increases the final size of the regenerated Leydig cell population above intact Vehicle + Sham controls, but not EDS + Sham animals, likely *via* paracrine interactions during the initial stages of regeneration. Whether this is a direct effect on Leydig stem/progenitors, or indirectly *via* activation of testicular macrophages requires further investigation.

## Materials and Methods

### Ethics

Animal experimentation was carried out in compliance with the Animals (Scientific Procedures) Act, 1986. All procedures were approved by University of Edinburgh Animal Welfare and Ethical Review Body and were conducted with licenced permission under UK Home Office regulations (project licence number 70/8804 held by Professor Lee B. Smith). Permission for the collection of tissue and subsequent research was granted in Edinburgh by the South East Scotland Research Ethics Committee (Reference: 10/S1103/45).

### Animals

Animals were maintained on a 12 hr light cycle (07:00–19:00) with controlled temperature (20–25 °C) and humidity (~55%). Soya-free chow and drinking water were available *ad libitum*. Male Wistar Kyoto rats (Wky/NCrl; n = 24) at 70–90 days of age were obtained from Charles River (Charles River Laboratories, Margate, UK). The animals were housed in groups of 4. Animals were acclimatised for at least one week prior to entry into experiments. The experimental overview is outlined in Fig. 2A.

### Leydig cell ablation

To ablate testicular Leydig cells, rats were treated with the alkylating agent ethane dimethanesulphonate (EDS), which has been widely used to induce Leydig cell-specific ablation and regeneration^27,63^. The EDS used in this study was a kind gift from Professor Peter O’Shaughnessy (Institute of Biodiversity, Animal Health and Comparative Medicine, University of Glasgow). On the day of injection, EDS was dissolved in sterile dimethyl sulfoxide DMSO (Hybri-Max^™^; Sigma-Aldrich, Dorset, UK) at 200 mg/mL. Immediately prior to injection, a 50 mg/mL working solution was prepared by diluting into DMSO:Water (1:3). Animals were dosed with 85 mg/kg EDS *via* intraperitoneal injection. Control animals were injected with an equivalent volume of vehicle (DMSO:Water).

### hAd-PSC culture and transplantation

Human adipose-derived perivascular stem cells (hAd-PSCs; CD146^pos^, CD34^neg^, CD31^neg^, CD45^neg^) used in these studies were isolated from the stromal vascular fraction of human lipoaspirates as previously described^25^. hAd-PSCs were maintained in expansion media (EM) consisting of DMEM GlutaMAX™ (Thermo Scientific, UK) supplemented with 20% (vol/vol) fetal bovine serum (FBS; Thermo Scientific, UK) and 1% (vol/vol) penicillin-streptomycin (Thermo Scientific, UK), at 37 °C with 5% CO_2_. One week prior to transplantation, sub-confluent cultures were either switched to a previously described differentiation-inducing media (DIM^11^), or maintained in EM. DIM consisted of phenol red-free DMEM/F-12 supplemented with 2% FBS, 1% penicillin-streptomycin (each from Thermo Scientific, UK), 10 ng/mL recombinant human platelet-derived growth factor beta (rhPDGF-BB), 1 ng/mL human luteinising hormone (hLH), 70 ng/mL recombinant human insulin-like growth factor-1 (rhIGF-1), 1 nM 3,3′,5-Triiodo-L-thyronine sodium salt (thyroid hormone, T3; UK) and 1X human ITS supplement (each from Sigma-Aldrich, UK). Cells were transplanted into the testis interstitium 4 days after EDS-mediated Leydig cell ablation. Briefly, testes were exposed, through a scrotal incision, under isoflurane anaesthesia. On average, approximately 1.8 (±0.62) million cells in 50 µL Opti-MEM™ (Thermo Scientific, UK) were injected under the tunica albuginea using a Micro-Fine™ + 0.3 mL (30 G) syringe (BD Bioscience, UK). A single sub-cutaneous injection of analgesic (Buprenorphine; 0.05 mg/kg) was administered prior to the animals regaining consciousness. A sham operation (injection of 50 µL of Opti-MEM™ only) was carried out on control animals.

### Tissue collection and processing

Blood samples were collected from the tail vein weekly between 08:00 and 11:00am for the duration of the experiment (5 weeks in total). Approximately 500 µL blood was collected into EDTA coated tubes (Greiner Bio-One, UK) and centrifuged at 1400 g for 10 mins to separate serum. Sera were stored at −80 °C. At the end of the experiment (35 days post EDS administration), body, testis and seminal vesicle weights were recorded. One testis from each animal was fixed in Bouin’s solution for 8 hrs. After fixation, testes were processed, embedded in paraffin wax and 5 µm sections prepared for histological analyses. The contralateral testis was frozen on dry ice and stored at −80 °C.

### Histological and immunohistochemistry

For general histological analysis, testis sections were stained with Haematoxylin and Eosin (H&E) following standard protocols. Immuno-labelling of Leydig cells was achieved by a chromogenic immunostaining procedure. Briefly, tissue sections were dewaxed and rehydrated following standard procedures. Heat-induced epitope retrieval was carried out by boiling tissue sections in 0.01 M citrate buffer (pH 6.0) using a Decloaking Chamber™ Pro (Biocare Medical, USA). Endogenous peroxidase activity was quenched by immersion of sections in 0.3% (v/v) hydrogen peroxide in Tris-buffered saline (TBS; pH 7.4) for 30 mins at room temperature. All incubations were carried out in a humidity chamber. Sections were subjected to 3 × 5 min washes in TBS between each incubation step. Binding of antibodies to non-specific epitopes was blocked by incubating sections with a blocking solution containing normal chicken serum (Biosera, Uckfield, UK) diluted 1:5 in TBS containing 5% (w/v) bovine serum albumin (BSA; Sigma-Aldrich Co. Ltd., UK). Sections were then incubated with primary antibody against the Leydig cell marker hydroxysteroid dehydrogenase 3-beta 1/6 (sc-30820, Lot #H0814; Santa Cruz Biotechnology, Germany) diluted 1:400 in blocking serum at 4 °C overnight. Next, sections were incubated with a biotin-conjugated secondary antibody (sc-2984; Santa Cruz Biotechnology, Germany), diluted 1:500 in blocking serum for 30 min at room temperature, followed by a 30 min incubation with streptavidin-horseradish peroxidase conjugate (SA-5004; Vector Labs, USA) diluted 1:1000 in TBS. Localisation of primary antibody was visualised by the addition of the chromogenic peroxidase substrate 3,3′-diaminobenzidine (DAB; Vector Labs, Peterborough, UK) according to the manufacturer’s instructions. Sections were counterstained with haematoxylin, dehydrated and mounted with glass cover slips. Control sections (i) from which the primary antibody had been omitted, (ii) primary antibody was replaced with isotype IgG (I9140, Lot 047K6095; Sigma-Aldrich, Dorset, UK) and (iii) sections from Leydig cell ablated rat testes incubated with primary antibody were included (Supplemental Fig. 4). Images were captured using an Axio Scan Z.1 slide scanner (Carl Zeiss Ltd, Welwyn Garden City, UK).

### Stereology

Leydig cells were identified in tissue sections by chromogenic immunostaining for HSD3B as described above. Leydig cell quantification was carried out using the stereology plug-in for Image-Pro plus 7.0 software (Media Cybernetics, UK) and a Zeiss Axio Imager A1 microscope (Carl Zeiss, UK) with a Qimaging QICAM Fast 1394 digital camera (Qimaging, Canada) and a Prior ProScan automated stage (Prior Scientific Instrument Ltd, UK). In brief, the ‘count’ function was used to score nuclei of immuno-positive cells at 630X magnification in a minimum of 70 random fields per section. Only fields containing ≥50% tissue area were scored. The resulting relative nuclear volume data were converted to absolute nuclear volume using testis weight. Mean nuclear volume was then measured from approximately 100 Leydig cell nuclei per section using the ‘nucleator’ function and used to calculate cell number per testis. This method has previously been shown to give comparable results to the dissector method for estimating cell number in the rat testis^64^.

### Hormone analysis

Circulating luteinising hormone (LH) was measured in serum using an in-house ELISA as previously described^65^. Standard curves were prepared with 8 different concentrations (10, 5, 2.5, 1, 0.5, 0.25 0.1 & 0 ng/mL). Samples, standards and controls were included in duplicate. Inter- and intra-assay CV were calculated from two controls of low and high LH in duplicate. The inter-assay CV for low and high pools respectively were 10.8 and 12.6%. The intra-assay CV were 7.6 and 10.7%. The lower limit of detection was calculated at 0.1 ng/mL. Testosterone was quantified in rat serum using an in house competitive ELISA. Briefly, 96-well plates (Greiner Bio-One GmbH, Germany) were coated with 100 µL of donkey anti rabbit IgG (Jackson ImmunoResearch Inc, USA) per well at a dilution of 1:500 in ELISA coating buffer (100 mM NaHCO_3_, pH 9.6). After overnight incubation at 4°C, plates were washed twice with wash buffer (0.05 M Tris-HCl, 0.05% Tween 20, pH 7.4) and incubated with 250 μL of blocking buffer (PBS pH 7.4 containing 0.5% BSA) for 1 hr at room temperature. Plates were then washed twice with wash buffer prior to the addition of standards, samples and controls (20 µL per well), followed by 80 µL of testosterone-HRP conjugate (Astra Biotech GmbH, Germany) at 1:20,000 in assay buffer (PBS pH 7.4 containing 0.1% BSA and 250 ng/mL Cortisol), followed by 50 μL of rabbit anti-testosterone-19 antibody (AMS Biotechnology, USA) at 1:200,000 in assay buffer. Plates were incubated at room temperature for 2 hr on an IKA^®^, Schuttler MTS4 plate shaker (IKA Labortechnik, Germany), then washed 5 times with assay wash buffer. Next, 120 µL of substrate solution (3,3,5,5-Tetramethylbenzidine; Millipore Corporation, USA) was added to each well and plates were incubated at room temperature, without shaking, in the dark, for 20 mins. The reaction was stopped by the addition of 80 µL stop solution (2N H_2_SO_4_; Sigma-Aldrich, UK). Finally, plates were read on a plate reader at 450 nm. Standard curves were prepared from 8 different concentrations (24.3, 8.1, 2.7, 0.9, 0.3, 0.1 0.03 & 0 ng/mL). Samples, standards and controls were included in duplicate. Inter- and intra-assay CV were calculated from two controls of low and high testosterone in duplicate. The inter-assay CV for low and high pools respectively were 13.6 and 7.2%. The intra-assay CV were 4.6 and 3.1%. The lower limit of detection was calculated at 0.2 ng/mL.

### Genomic PCR

Genomic DNA was isolated from frozen testes using the GeneJET Genomic DNA Purification Kit (Thermo Scientific, UK) and interrogated for the presence/absence of human-specific genomic DNA by PCR as described by Caspi, *et al*.^66^. Multiplex PCR amplification of α-satellite region of human chromosome 17 (*HUMSAT17A*) and rat beta actin (*Actb*; as an internal control) was performed using the Type-it Mutation Detect PCR Kit (QIAGEN Ltd., UK). The primer sequences were *HUMSAT17A-*FWD gggataatttcagctgactaaacag, *HUMSAT17A-*REV gtgtttcatagctgctctttcca and *Actb-*FWD tgtgttcttgccctctttgc, *Actb-*REV caggaaggaaggctggaaga. PCR products were analysed using the QIAxcel capillary electrophoresis system (QIAGEN Ltd., UK). Genomic DNA isolated directly from EM and DIM cultured Ad-PSCs was included as a positive control. A template negative control (water instead of gDNA) was included to ensure reagents were free of contaminating gDNA.

### Quantitative RT-PCR

Gene expression (*mRNA*) analyses were carried out as previously described^67^ with minor modifications. In brief, total RNA was extracted from frozen testis tissues or from hAd-PSC cultures using the RNeasy^®^ Mini kit (QIAGEN Ltd., UK) following the manufacturer’s instructions with the inclusion of an on column DNase (QIAGEN Ltd., UK) treatment step. For testis tissue, a *Luciferase* RNA (75 ng/testis; Promega, UK) was added to each sample at the start of the extraction as an external control. RNA concentrations were estimated using a NanoDrop One spectrophotometer (Thermo Scientific, UK) with a 260:280 absorbance ratio of ~2.0 indicative suitably pure RNA. cDNA was prepared from up to 2 μg total RNA using the SuperScript^®^ VILO^™^ cDNA Synthesis Kit (Life Technologies, UK) following the manufacturer’s instructions and a reverse transcriptase negative (-RT) control was included to ensure reagents were free of nucleic acid contamination. Real-time PCR was carried using the ABI Prism 7900HT Real-Time PCR System (Applied Biosystems, UK) and the Roche Universal ProbeLibrary (Roche, UK) in 384-well format. Assays were designed using the online Universal Probe Library Assay Design Centre (Roche; https://lifescience.roche.com/en_gb/articles/Universal-ProbeLibrary-System-Assay-Design.html). Assays were designed spanning an intron to avoid the amplification of potential genomic DNA contaminants. Additionally, an –RT and a template negative control (water instead of cDNA) were included for each assay to ensure reagents were free of contaminating nucleic acid. Details of each assay are listed in Table 1. The external *Luciferase* control was assayed using gcacatatcgaggtgaacatcac and gccaaccgaacggacattt forward and reverse primers respectively and, a 5′NED labelled probe (tacgcggaatacttc). Data were analysed using the ΔΔCt method, with the expression of each gene related to the external *Luciferase* control or internal *ACTB* control (Roche, UK) for rat testis and hAD-PSC cDNAs respectively. Amplification efficiency of qRT-PCR assays was first determined using a serial dilution of control cDNA. Assays with an efficiency between 90 and 105% were considered suitably efficient for ΔΔCt analyses.

**Table 1:** Details of qRT-PCR Assays.

Gene	Forward Primer	Reverse Primer	Probe
*Lhcgr*	CTGGAGAAGATGCACAGTGG	CTGCAATTTGGTGGAAGAAATA	107
*Star*	TCACGTGGCTGCTCAGTATT	CTGGGTCTGTGATAAGACTTGGT	83
*Cyp11a1*	ACCTATTCCGCTTTGCCTTT	CACGATCTCCTCCAACATCC	9
*Hsd3b1*	AAGGCCAAGGTGACAATGTT	TGTGAGACATCAATGACAGCAG	105
*Cyp17a1*	CATCCCCCACAAGGCTAAC	TGTGTCCTTGGGGACAGTAAA	67
*Hsd17b3*	ATGTCACGATTGGAGCTGAA	AAGGAATCAGGTTCAGAATTATCG	5
*STAR*	GGCATCCTTAGCAACCAAGA	TCTGGGACCACTTTACTCATCA	18
*CYP11A1*	GATGACCTGTTCCGCTTTG	CCTCGGGGTTCACTACTTCC	89
*CYP17A1*	CTATGCTCATCCCCCACAAG	CCTTGTCCACAGCAAACTCA	67
*HSD17B3*	AACTTGCAGGCTTAGAAATTGG	GGTGCGTTCAGGAAATGG	7

### Statistical analyses

Data are expressed as the mean ± S.E.M. Statistical analyses were carried out in GraphPad Prism 7.02 for Microsoft Windows (GraphPad Software Inc., USA). In the first instance, data distribution was assessed using the Shapiro-Wilk normality test. Differences between two experimental groups were identified using an unpaired, two tailed, *t*-test. Differences between more than two experimental groups were identified using either a one-way analysis of variance (1-way ANOVA) or a Kruskal –Wallis test. In the case of 1-way ANOVAs, Tukey’s post hoc analysis was used to compare means between groups. For Kruskal-Wallis, means were compared using Dunn’s post hoc test. A *p*-value of ≤0.05 was considered statistically significant.

## Supplementary information

Curley et al hAd-PSCs Supplemetary Info
