# Supplementary material for: Human Adipose-derived Pericytes Display Steroidogenic Lineage Potential in Vitro and Influence Leydig Cell Regeneration in Vivo in Rats
Source: Sci Rep. 2019 Oct 21;9:15037. doi: 10.1038/s41598-019-50855-0 (PMC6803635; doi:10.1038/s41598-019-50855-0)
Supplement: Supplementary file 1 — Curley et al hAd-PSCs Supplemetary Info [file 41598_2019_50855_MOESM1_ESM.pdf]

Human Adipose-derived Pericytes Display Steroidogenic Lineage Potential *in Vitro* and Influence Leydig Cell Regeneration *in Vivo* in Rats

Michael Curley[1], Zaniah N. Gonzalez[2], Laura Milne[1], Patrick Hadoke[3], Ian Handel[4], Bruno Péault[2, 5] and Lee B. Smith[1, 6, †]

[1] MRC Centre for Reproductive Health, University of Edinburgh, The Queen's Medical Research Institute, 47 Little France Crescent, Edinburgh, EH16 4TJ, United Kingdom.

[2] MRC Centre for Regenerative Medicine, University of Edinburgh, Edinburgh Bioquarter, 5 Little France Drive, EH16 4UU, United Kingdom.

[3] The British Heart Foundation Centre for Cardiovascular Science, University of Edinburgh, The Queen's Medical Research Institute, Edinburgh, EH16 4TJ, United Kingdom.

[4] The Roslin Institute and Royal (Dick) School of Veterinary Studies, University of Edinburgh, EH25 9RG.

[5] Department of Orthopaedic Surgery and Broad Stem Cell Center, University of California at Los Angeles, 615 Charles E Young Dr S, Los Angeles, CA 90095, USA.

[6] School of Environmental and Life Sciences, University of Newcastle, Callaghan, NSW 2308, Australia.

[†] Correspondence: Professor Lee Smith, MRC Centre for Reproductive Health, University of Edinburgh, The Queen's Medical Research Institute, 47 Little France Crescent, Edinburgh, EH16 4TJ, United Kingdom.

e-mail: lee.smith@ed.ac.uk

Supplementary Information

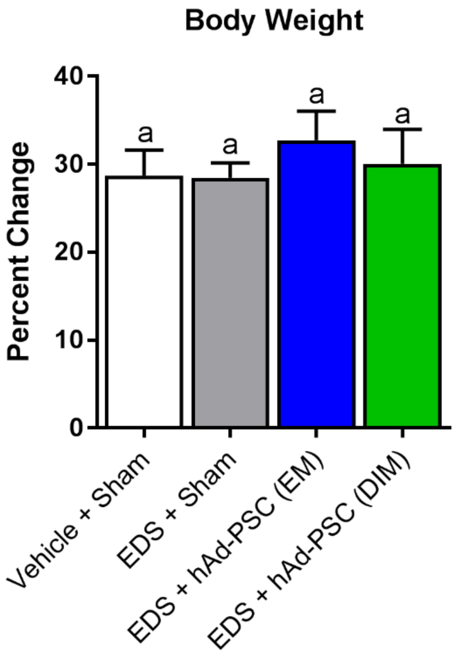

Supplemental Figure 1. No difference in body weight was noted in experimental animals. Body weight changes were similar between Vehicle + Sham controls and Leydig cell ablated animals with or without transplantation of EM or DIM cultured hAd-PSCs (1-way ANOVA). Tukey's post-hoc analysis was used to compare means between groups where a shared letter denotes no significant difference. Data presented are mean ± SEM from n=5-6 separate animals per group.

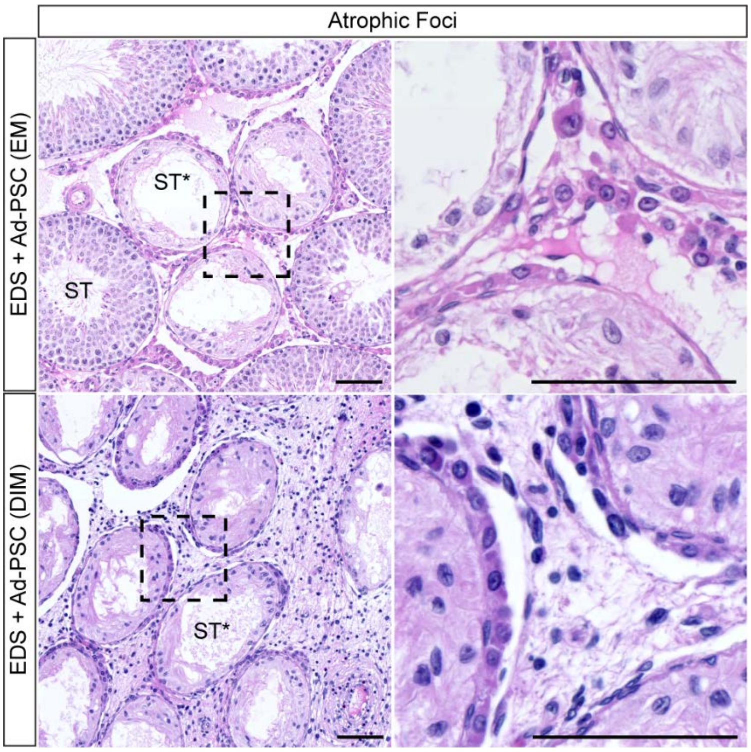

Supplemental Figure 2. Regions containing atrophic tubules. Occasionally, regions containing atrophic tubules (ST*) and possible inflammatory cell infiltrates were noted both in EM and DIM hAd-PSC-transplanted testes. Scale = 100µm.

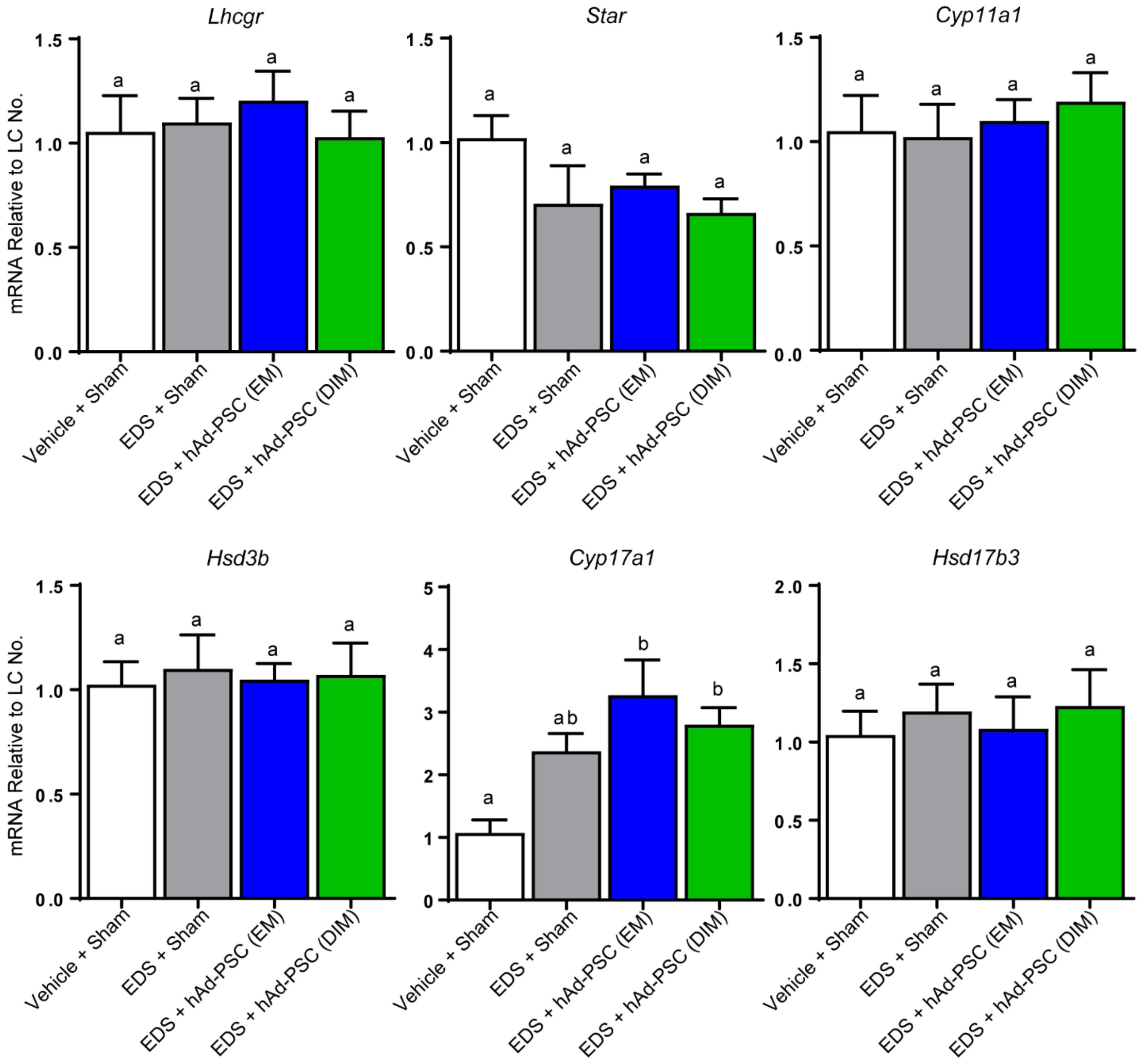

Supplemental Figure 3. Steroidogenic *mRNA* expression 'per Leydig cell'. No difference in *mRNA* expression levels (relative to Leydig cell number) of **l**uteinising hormone/chorionic gonadotropin receptor (*Lhcgr*); steroidogenic acute regulatory protein (*Star*); P450 cholesterol side-chain cleavage enzyme (*Cyp11a1*); hydroxysteroid dehydrogenase 3-beta (*Hsd3b*); or hydroxysteroid dehydrogenase 17-beta type 3 (*Hsd17b3*) was noted between experimental groups and Vehicle + Sham controls (1-way ANOVA). However, expression of 17α-hydroxylase, 17,20-lyase (*Cyp17a1*) was significantly increased both in hAd-PSC (EM) and in hAd-PSC (DIM) groups (1-way ANOVA; $p = 0.0027$). Tukey's post-hoc analysis was used to compare means between groups where a shared letter denotes no significant difference. Data presented are mean ± SEM from n=5-6 separate animals per group.

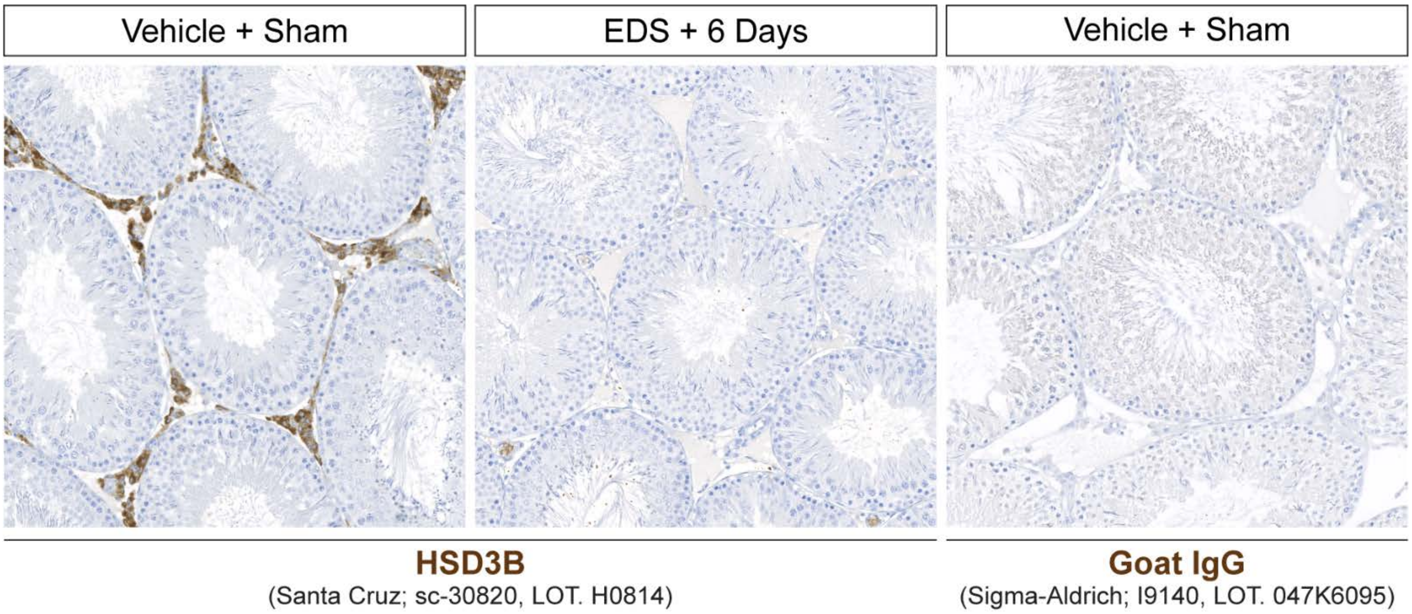

Supplemental Figure 4. Immunohistochemistry primary antibody controls. Sections of Vehicle + Sham control testis incubated with primary antibody (positive control, left pane) shows positive HSD3B staining (brown) in interstitial cells. No staining is observed in sections of rat testis incubated with primary antibody 6 days following EDS-mediated Leydig cell ablation (middle pane). No staining is observed in sections of Vehicle + Sham control testes incubated with isotype IgG (right pane; I9140, Lot 047K6095; Sigma-Aldrich, Dorset, UK).
